# Supplementary material for: Improving integrative 3D modeling into low‐ to medium‐resolution electron microscopy structures with evolutionary couplings
Source: Protein Sci. 2021 Apr 9;30(5):1006–21. doi: 10.1002/pro.4067 (PMC8040867; doi:10.1002/pro.4067)
Supplement: Supplementary file 1 — Appendix S1: Supporting information [file PRO-30-1006-s001.pdf]

Supplemental Material

Improving integrative 3D modeling into low- to medium- resolution EM structures with evolutionary couplings

Caitlyn L. McCafferty, David W. Taylor, Edward M. Marcotte

PDB	Complex	Organism	Stoichiometry
1FFT	Ubiquinol oxidase	*E. coli*	ABC
1L0V	Quinol-fumerate reductase	*E. coli*	ABCD
5D0O	BamABCDE	*E. coli*	ABCDE
5D0Q	BamACDE	*E. coli*	ABCD
5MRW	KdpFABC	*E. coli*	ABC

Supplemental Table 1. Complexes modeled.

Protein Uniprot ID	Number of Sequences	Neff
P0ABI8	96075	11603.376
P0ABJ1	71670	17941.866
P0ABJ3	63525	17339.788
P00363	75181	19342.215
P0A8Q0	2438	751.050
P0A8Q3	2261	671.708
P0AC47	35803	9159.588
P03959	22107	4586.527
P03960	21488	3570.625
P03961	18470	6418.302
P0A903	3264	690.742
P0A937	9155	3605.051
P0AC02	16947	7477.941
P0A940	26541	9087.179
P77774	12200	4846.372

Supplemental Table 2. Number of sequences used in co-evolutionary analysis.

Protein Complex PDB	10 Å Cutoff Cα-RMSD	No Couplings Cα-RMSD
1FFT	4.522	768.562
5MRW	21.915	57.136
5D0Q	25.453	762.919
5D0O	30.630	431.027

Supplemental Table 3. Cα-RMSD between the central cluster model with and without evolutionary couplings restraint for X-ray crystal structures from the Protein Data Bank.

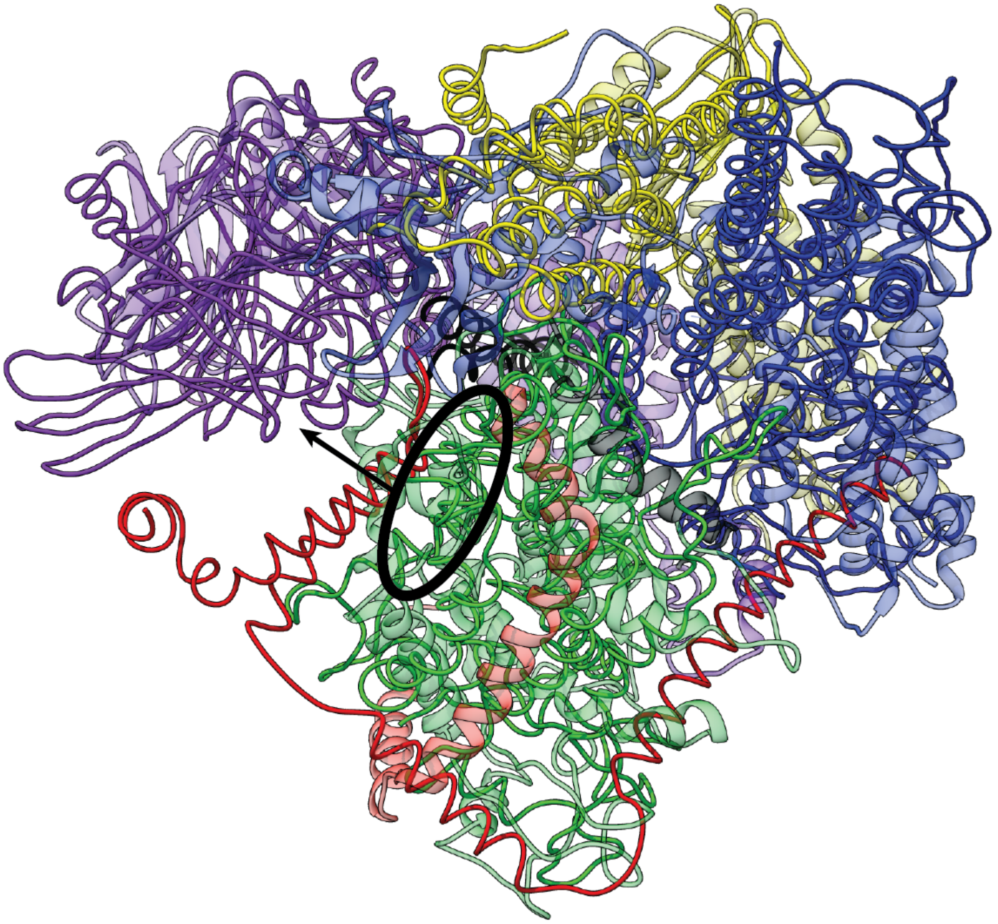

Supplemental Figure 1. Integrative model of the bacterial holo-translocon superimposed on the published model (PDB: 5MG3). Crosslinked region of SECY is circled.

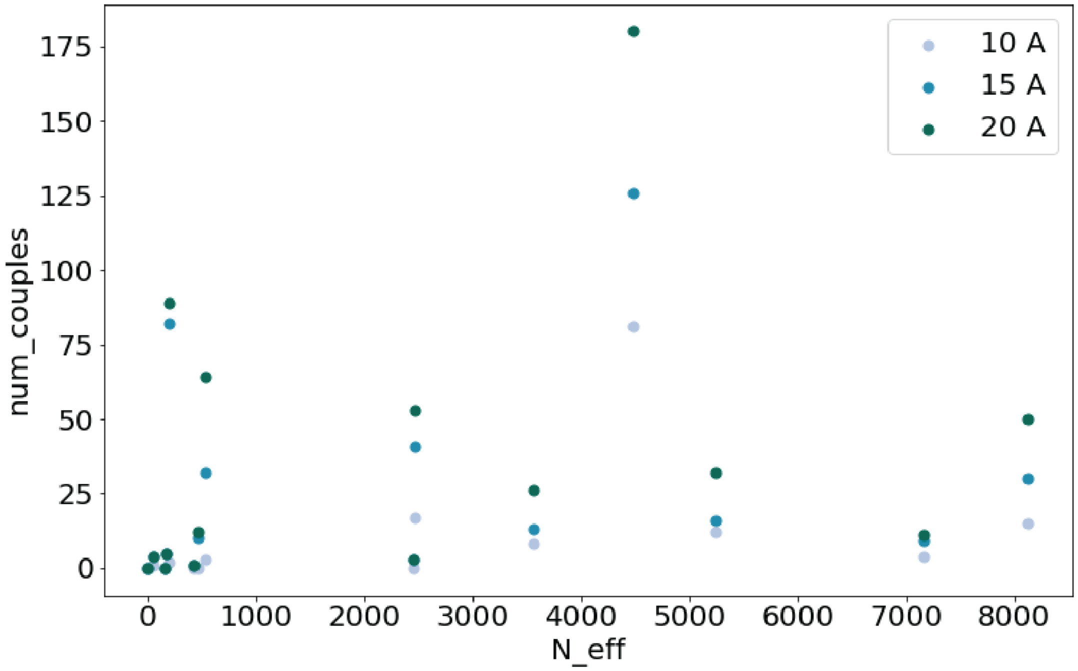

Supplemental Figure 2. Number of effective sequences between protein subunits vs the number of couplings using the internal calibration method between the same subunit pair.
